# Identifying Quantitative Trait Loci (QTLs) and Developing Diagnostic Markers Linked to Orange Rust Resistance in Sugarcane (*Saccharum* spp.)

**DOI:** 10.3389/fpls.2018.00350

**Published:** 2018-03-19

**Authors:** Xiping Yang, Md. S. Islam, Sushma Sood, Stephanie Maya, Erik A. Hanson, Jack Comstock, Jianping Wang

**Affiliations:** ^1^Department of Agronomy, Genetics Institute, University of Florida, Gainesville, FL, United States; ^2^Sugarcane Field Station, United States Department of Agriculture-Agricultural Research Service, Canal Point, FL, United States; ^3^Key Laboratory of Genetics, Breeding and Multiple Utilization of Corps, Center for Genomics and Biotechnology, Ministry of Education and Fujian Provincial Key Laboratory of Haixia Applied Plant Systems Biology, Fujian Agriculture and Forestry University, Fujian, China

**Keywords:** orange rust disease, sugarcane, *Saccharum* spp., quantitative trait locus (QTL), marker-assisted selection (MAS)

## Abstract

Sugarcane (*Saccharum* spp.) is an important economic crop, contributing up to 80% of table sugar used in the world and has become a promising feedstock for biofuel production. Sugarcane production has been threatened by many diseases, and fungicide applications for disease control have been opted out for sustainable agriculture. Orange rust is one of the major diseases impacting sugarcane production worldwide. Identifying quantitative trait loci (QTLs) and developing diagnostic markers are valuable for breeding programs to expedite release of superior sugarcane cultivars for disease control. In this study, an F_1_ segregating population derived from a cross between two hybrid sugarcane clones, CP95-1039 and CP88-1762, was evaluated for orange rust resistance in replicated trails. Three QTLs controlling orange rust resistance in sugarcane (qORR109, qORR4 and qORR102) were identified for the first time ever, which can explain 58, 12 and 8% of the phenotypic variation, separately. We also characterized 1,574 sugarcane putative resistance (*R*) genes. These sugarcane putative *R* genes and simple sequence repeats in the QTL intervals were further used to develop diagnostic markers for marker-assisted selection of orange rust resistance. A PCR-based *Resistance* gene-derived maker, G1 was developed, which showed significant association with orange rust resistance. The putative QTLs and marker developed in this study can be effectively utilized in sugarcane breeding programs to facilitate the selection process, thus contributing to the sustainable agriculture for orange rust disease control.

## Introduction

Sugarcane (*Saccharum* spp.) is one of the most important economic crops, cultivated on ∼27.1 million hectares in over 100 countries with a worldwide harvest of 1.9 billion metric tons and a gross production value of $81.5 billion (FAO, 2013, 2014). As the most important sugar resource, sugarcane contributes up to 80% of sugar production in the world. Additionally, sugarcane accounts for ∼60% of global bio-ethanol production, with an energy output-to-input ratio five times higher than that of maize (Waclawovsky et al., 2010; Dahlquist, 2013). Nevertheless, like many other crops, sugarcane production has been vulnerable by many diseases.

Orange rust, caused by *Puccinia kuehnii*, was first reported in Australia more than 100 years ago; however, this disease only began to draw attention due to its devastating epidemic in 2000 on a major Australian sugarcane cultivar Q124 (Braithwaite et al., 2009). Orange rust was first identified in the Western Hemisphere, specifically in Florida, United States, in 2007 (Comstock et al., 2008) and shortly after in Guatemala (Ovalle et al., 2008), Mexico, El Salvador, Panama (Flores et al., 2009), Costa Rica, Nicaragua (Chavarria et al., 2009), Brazil (Barbasso et al., 2010), Colombia (Cadavid et al., 2012), and recently Ecuador (Garcés et al., 2014). The disease immediately became an emerging threat to sugarcane production and breeding programs since its discovery (Comstock et al., 2008, 2010), which can cause up to 50% cane yield loss in its susceptible hosts (Magarey et al., 2011).

In the United States, Florida is the top sugarcane producer for sugar production with ∼28.0 million tons of cane and a gross value of $909.7 million annually (FAO, 2014). The majority of commercial cultivars grown in Florida and the parental germplasm used in the sugarcane breeding program are susceptible to the orange rust (Comstock et al., 2010), marking this disease a major concern in sugarcane industry. Application of fungicides has proven to increase sugar production from 7.9 to 26% on CP80-1743 in commercial fields (Comstock et al., 2010). However, fungicide treatments can lead to input cost increasing as well as potential environmental problems, which is against the principles advocated in sustainable agriculture (Lichtfouse et al., 2009). Therefore, developing and utilizing resistant cultivars is undoubtedly the favorable approach for sugarcane breeders and farmers for the disease control.

Screening for disease resistant sugarcane breeding materials can be performed by evaluating plant reactions both in the field and/or in greenhouse after rust spore inoculation artificially. The phenotype-based selection is direct and efficient when plants are inoculated with viable inoculum at the right growth stages in an environment favoring disease development. Unfortunately, selection based on phenotyping is time-consuming, labor-intensive, and environment-dependent due to requirement of large space, inoculation process, and possible escapes if favorable conditions or plant developmental stages are not achieved during inoculation. Marker-assisted selection (MAS) of resistant materials is a desirable alternative method, in which resistant individuals can be selected in the laboratory in less than 24 h by testing the presence of molecular markers linked to disease resistance. The MAS depends on reliable markers that are tightly linked to genes or genomic regions controlling disease resistance through quantitative trait loci (QTL) mapping and association study. Though sugarcane is a complex auto-polyploid species with large genome size, several disease resistance loci have been identified, such as *Bru*1 (Daugrois et al., 1996) and *Bru*2 (Raboin et al., 2006) for brown rust resistance, four DNA markers for pachymetra root rot and leaf scald resistance, five for Fiji leaf gall resistance, and 11 for smut resistance (Wei et al., 2006), a major QTL for yellow spot resistance (Aljanabi et al., 2007), a major quantitative trait allele for the *Sugarcane yellow leaf* resistance (Costet et al., 2012b) were identified in sugarcane. Once identified and validated, these molecular markers could be used in MAS to quickly identify and select desirable resistant resources or progeny, though no disease resistance gene has been cloned in sugarcane yet. For example, since the discovery of *Bru*1 and the identification of its co-segregating markers (R12H16 and 9O20-F4) (Costet et al., 2012a), the diagnostic markers have widely been used in sugarcane breeding programs. In Florida sugarcane breeding program, the markers for *Bru*1 are being utilized to evaluate brown rust resistance in sugarcane germplasm and hybrid clones (Glynn et al., 2013). It was estimated that 27% of the clones used for crossing (a total of 1027) contained *Bru*1, and the frequency of *Bru*1 in sugarcane clones increased from 15% (1975–1985) to 47% (2002–2012) after brown rust was introduced to Florida.

Although reliable molecular markers are of great value for controlling disease effectively in a way fitting the goals of sustainable agriculture, so far none has been reported to be linked to orange rust resistance in sugarcane. The objectives of this study were (1) to phenotypically evaluate the orange rust resistance reaction in a mapping population; (2) to map QTLs controlling orange rust resistance in sugarcane; and (3) to develop diagnostic markers for MAS of orange rust resistance in sugarcane. The putative QTLs and markers developed in this study can be effectively utilized in sugarcane breeding programs to expedite release of resistant cultivars, thus contributing to sustainable agriculture for orange rust disease control.

## Materials and Methods

### Plant Materials

The sugarcane mapping population comprised of 173 F_1_ progeny that were derived from a cross between sugarcane clones CP95-1039 and CP88-1762, which were developed by the United States Department of Agriculture (USDA), Agricultural Research Service (ARS) Sugarcane Field Station at Canal Point, FL, United States. Individuals of the progeny were clonally propagated for leaf sampling and phenotype evaluation. The whole population was genotyped using genotyping by sequencing (GBS), and sequence variations were called according to Yang et al. (2017a). The GBS libraries and sequencing were performed at the Institute of Genomic Diversity, Cornell University following the optimized protocol (Elshire et al., 2011) with a rare cutting restriction enzyme *Pst*I (CTGCAG) for library construction and a 96-plex (95 DNA samples and one negative control) for sequencing on the Illumina HiSeq 2000 platform (Yang et al., 2017b). The cleaned reads were aligned to sorghum genome v3.0 (Paterson et al., 2009) for calling single nucleotide polymorphisms (SNPs) using seven different SNP callers described by Yang et al. (2017b). Only the single dose markers were used for genetic map construction using Joinmap 4.0 (Van Ooijen, 2006). Two high density genetic maps were constructed with one for CP95-1039 including 2,453 markers and a total length of 4224.4 cM and the other for CP88-1762 including 2,154 markers and a total length of 4373.2 cM (Yang et al., 2017b). For validating the diagnostic marker developed in this study, a diversity panel with 165 sugarcane clones derived from multiple crosses in multiple years (Supplementary Table S1) was used.

### Evaluation of Orange Rust Resistance

The whole F_1_ mapping population along with the two parental clones, CP95-1039 and CP88-1762, were used in orange rust resistance evaluation. CP95-1039 is resistant to orange rust, whereas CP88-1762 is susceptible to the disease (Supplementary Figure S1). Rust resistance evaluation of this population was conducted using artificial inoculation methods under both field and greenhouse conditions.

The inoculum preparation and inoculation were performed following the protocol described by Sood et al. (2009). Briefly, sugarcane orange rust urediniospores were collected by vacuuming the abaxial side of symptomatic leaves since *P. kuehnii* is biotrophic and difficult to culture in medium. A large number of leaves diagnosed with orange rust disease symptoms from multiple highly susceptible cultivars were used to collect enough inoculum for this experiment. Freshly collected urediniospores were used as inoculum or stored at -70°C. Rust spores were suspended in sterile distilled water containing 0.1% (V/V) Tween-20 and 0.002% 1-nonanol with an adjusted concentration of 10^4^ urediniospores/ml.

For the field experiment, seedlings from the mapping population grown in the greenhouse were transplanted in the field on August 2010 for population establishment at the USDA-ARS Sugarcane Field Station, Canal Point, FL, United States. The planting canes were trimmed above the soil in February 2011 for first ratoon regrowth. The inoculum was applied to three stalks of each genotype when the sugarcane seedlings were 2 months old after transplanting in October 2010, and when the regrown ratoon plants were 2 and 3 months old after cutting in April 2011 and May 2011, respectively. To infect plants, one-third of the tips of the uppermost leaves were cut off as a marker, and a 0.5-ml aliquot of the spore suspension was deposited inside the leaf whorl of the marked stalks. Three weeks post-inoculation, any rust signs or symptoms were recorded from the newly emerged leaf following a 0–4 scale (Sood et al., 2009). A score of 0 indicated that the plants were asymptomatic. A score of 1 corresponded to the presence of some chlorotic flecks without any pathogen spores on the leaves. A score of 2 indicated that leaves had some brown discoloration, but no pustules or sporulation. A score of 3 corresponded to the presence of ≤10 pustules and sporulation on the leaves. A score of 4 corresponded to >10 pustules with massive sporulation. The inoculation on plant cane was scored one time, while each inoculation on ratoons was scored by two researchers independently. Five observations were conducted after artificial inoculation in the field; one was on the plant cane and the other four were on ratoons.

For the greenhouse experiment, individuals of the mapping population were clonally propagated with four plants per sugarcane clone in 46 cm × 38 cm × 10 cm flats filled with Miracle-Gro^®^ Potting Mix (Scotts Miracle-Gro^®^ Company, Marysville, OH, United States). Scott’s Osmocote Classic 14-14-14 fertilizer (Scotts-Sierra, Marysville, OH, United States) and daily irrigation were applied to maintain healthy plants. Each flat contained four genotypes, with four stalks for each genotype. The inoculum (prepared in the same way as described above) was applied to three healthy stalks (4 months old) with a complete leaf whorl developed. Three weeks after inoculation, any rust signs or symptoms were scored from the newly emerged leaf following the 0 (no disease present) to 4 (highly susceptible) scale (Sood et al., 2009). Artificial inoculation experiments were conducted under greenhouse conditions in March 2014, November 2014 and May 2015, respectively. The first greenhouse inoculation experiment was performed on plant cane, whereas the other two inoculation experiments were on ratoons. The inoculation and scoring of rust symptoms in the greenhouse were the same as described above. Therefore, three observations were conducted after artificial inoculation in the greenhouse.

### Data Analysis and QTL Mapping

A pairwise Pearson correlation among scores of eight observations of orange rust resistance was calculated using the ‘Hmisc’ package in R3.0.2 (R Development Core Team, 2013; Harrell, 2018). Analysis of variance was performed using the ‘nlme’ package in R (Pinheiro et al., 2009). Broad sense heritability (H^2^) was estimated using the formula below:

$${\text{H}}^{\text{2}}\text{=}\frac{\delta_{\text{9}}^{\text{2}}}{\delta_{\text{9}}^{\text{2}}\text{+}\delta_{\text{e}}^{\text{2}}}$$

where $\delta_{9}^{2}$ and $\delta_{9}^{2}$ are the genetic variance of progeny lines, and the error variance, respectively.

The eight observations of orange rust resistance were manually checked for ‘escape’ inoculation (progeny with a score of two or more after inoculation were considered as ‘susceptible,’ and then only observations with scores higher than two were used to calculate average scores), and average scores were used for QTL analysis. A pseudo-testcross strategy was employed for QTL analysis using WinQTLCart 2.5 (Wang et al., 2012). Composite interval mapping (CIM) was performed using forward and backward stepwise regressions to select markers as cofactors with 10 cM window size and 1 cM walking speed in WinQTLCart 2.5 (Wang et al., 2012). The QTL nomenclature was used according to McCouch (2008).

### Identification of Sugarcane Putative *R* Genes

Five sugarcane transcript sequence databases (**Table 1**), including a total of two million sugarcane transcripts, were used to identify sugarcane transcripts having homologous genes in the sorghum genome. Redundant transcripts were removed by using cdhit/4.6 (sequence identity threshold 0.95) to reduce subsequently computational and manual efforts (Li and Godzik, 2006; Fu et al., 2012). The non-redundant sugarcane transcripts and their corresponding sorghum orthologs (Paterson et al., 2009) were identified and verified by using reciprocal BLASTN. The sugarcane transcripts with corresponding sorghum orthologs were used for subsequent analysis.

**Table 1:** Sugarcane transcript database sources used for identification of sugarcane putative resistance genes.

	Number of sequences	Sum of nucleotides	Average length
NCBI UniGene^1^	220,997	136,000,000	615
SOGI UniGene^2^	490,240	361,700,000	738
In-house RNA-Seq dataset 1	102,944	69,590,000	676
In-house RNA-Seq dataset 2	1,080,060	441,200,000	408
In-house RNA-Seq dataset 3	101,080	73,280,000	725
Total	1,995,321	1,081,770,000	542
Unique transcripts	700,194	345,243,774	493

*^*1*^ftp://ftp.ncbi.nlm.nih.gov/repository/UniGene/Saccharum_officinarum/.*

*^*2*^ftp://occams.dfci.harvard.edu/pub/bio/tgi/data/Saccharum_officinarum/.*

Sugarcane putative *R* genes were identified by two complementary methods. First, BLASTN of sugarcane transcripts with corresponding sorghum orthologs was performed against known plant *R* genes, which included 112 reference resistance genes with experimental support from the Plant Resistance Genes database (PRGdb)^1^ (Sanseverino et al., 2013). Second, InterProScan 5.0 was used to characterize sugarcane nucleotide-binding site (NBS)–leucine-rich repeat (LRR) genes from the sugarcane transcripts with corresponding sorghum orthologs following the procedures described previously (Jones et al., 2014; Yang and Wang, 2016). All bioinformatics analyses were performed locally at the University of Florida High Performance Computing Center using command lines.

### Development of PCR-Based Markers for Orange Rust Resistance Selection

Sugarcane simple sequence repeats (SSRs) sequences were collected from published literatures and aligned to sorghum genome 3.0 (Liu et al., unpublished data). Primers for putative *R* genes were designed using Primer3.^2^ Primers across introns based on the sorghum gene models were selected. Only primers uniquely aligned to the sorghum genome and aligned to sugarcane transcripts with no more than two mismatches were selected for synthesis (Invitrogen, Carlsbad, CA, United States). All the SSRs and gene markers were first screened using two parental clones, CP95-1039 (resistant to orange rust) and CP88-1762 (susceptible to orange rust). A touchdown PCR program was used as described by Li et al. (2011) for amplification. In brief, the reaction mixture was incubated at 95°C for 5 min, then five cycles of 60 s of denaturing at 96°C, 5 min of annealing at 68°C with a decrease of 2°C in each subsequent cycle, and 1 min of extension at 72°C; For another five cycles, the annealing temperature started at 58°C for 2 min with a decrease of 2°C for each subsequent cycle; PCR continued through an additional 25 cycles of 60 s at 96°C, 1 min at 50°C, and 1 min at 72°C with a final extension at 72°C for 5 min. PCR products were run on a 1% agarose gel in a horizontal electrophoresis apparatus (Bio-Rad Laboratories, Hercules, CA, United States). Polymorphic markers were used to genotype the mapping population of 173 F_1_ progeny derived from the cross between CP95-1039 and CP88-1762. Candidate diagnostic markers (PCR-based) were further genotyped in a diversity panel with 165 sugarcane clones derived from multiple crosses in multiple years (Supplementary Table S1). These 165 sugarcane clones were also screened phenotypically for orange rust resistance in the field following the protocol described above in the evaluation of orange rust resistance section. The Student *t*-tests of each marker were conducted for mean orange rust scores by using functions in Excel 2010 (Microsoft Corp., Redmond, WA, United States).

Based on artificial inoculation data, progeny with scores of 0, 1, and 2, that were not sporulating, were categorized as phenotypic ‘resistant,’ and progeny with scores of 3 and 4, that were sporulating, were categorized as phenotypic ‘susceptible.’ The 173 progeny were considered as ‘resistant’ or ‘susceptible’ based on sporulation for marker evaluation. Based on the closest flanking SNP markers to the significant QTLs, each individual in the whole population was characterized as genotypic ‘resistant’ or genotypic ‘susceptible.’ Selection accuracy was defined as the percentage of individuals which were both phenotypic and genotypic resistant among the total number of genotypic resistant individuals. Selection efficiency was defined as the percentage of individuals which were both phenotypic and genotypic resistant among the total number of phenotypic resistant individuals in the mapping population. Selection accuracy and selection efficiency were calculated following formula:

Selection accuracy = (the number of both phenotypic and genotypic resistant individuals/the number of genotypic resistant individuals) ^∗^ 100.
Selection efficiency = (the number of both phenotypic and genotypic resistant individuals/the number of phenotypic resistant individuals in the mapping population) ^∗^ 100.

## Results

### Phenotypic Data Analysis

**FIGURE 1:**
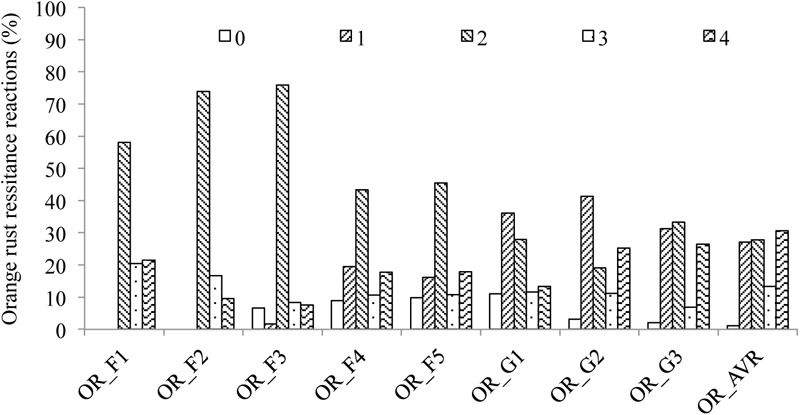
Distribution of orange rust (OR) resistance reactions in the mapping population across multiple trials observations. F, Field; G, Greenhouse; AVR, average.

Orange rust resistance reactions of the mapping population showed a nearly normal distribution (**Figure 1**), supporting orange rust resistance segregating in this bi-parental mapping population is controlled by multiple genes/alleles. Orange rust resistance reactions of artificial inoculations were highly correlated (*P* < 0.0001) with an average coefficient of 0.64 (**Table 2**). Heritability of orange rust resistance was 0.67, indicating that the disease resistance in the mapping population was largely controlled by genetic factors. In this study, the mean scores of the eight rust resistance observations were used for subsequent QTL analysis, which can reduce discrepancy caused by experimental errors in single artificial inoculations.

**Table 2:** Pearson’s correlation coefficients among rust resistance reactions on the F_1_ population derived from the cross CP95-1039 × CP88-1762.

	OR_F2	OR_F3	OR_F4	OR_F5	OR_G1	OR_G2	OR_G3	OR_mean
OR_F1	0.74^∗∗∗^	0.66^∗∗∗^	0.71^∗∗∗^	0.69^∗∗∗^	0.69^∗∗∗^	0.47^∗∗∗^	0.61^∗∗∗^	0.76^∗∗∗^
OR_F2		0.77^∗∗∗^	0.79^∗∗∗^	0.77^∗∗∗^	0.60^∗∗∗^	0.47^∗∗∗^	0.56^∗∗∗^	0.77^∗∗∗^
OR_F3			0.70^∗∗∗^	0.68^∗∗∗^	0.57^∗∗∗^	0.37^∗∗∗^	0.47^∗∗∗^	0.62^∗∗∗^
OR_F4				0.93^∗∗∗^	0.65^∗∗∗^	0.33^∗∗^	0.50^∗∗∗^	0.79^∗∗∗^
OR_F5					0.65^∗∗∗^	0.33^∗∗^	0.47^∗∗∗^	0.78^∗∗∗^
OR_G1						0.60^∗∗∗^	0.65^∗∗∗^	0.74^∗∗∗^
OR_G2							0.55^∗∗∗^	0.73^∗∗∗^
OR_G3								0.75^∗∗∗^

*^∗∗^*P* < 0.001; ^∗∗∗^*P* < 0.0001; F, Field; G, Greenhouse.*

### QTL Analysis

**FIGURE 2:**
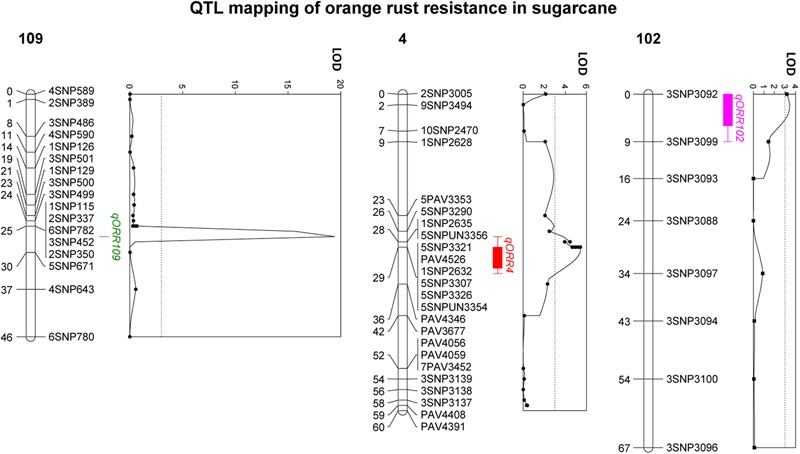
Logarithm of odds (LOD) score plots of the quantitative trait loci (QTLs) for sugarcane orange rust resistance in the regions harboring the three identified QTL clusters.

High density genetic maps of both parental clones (Yang et al., 2017b) were used for detecting QTLs employing WinQTLCart 2.5 (Wang et al., 2012). Three QTLs controlling orange rust resistance in sugarcane, qORR109, qORR4 and qORR102, were detected in the mapping population (**Figure 2** and **Table 3**). Of the three QTLs, one was located on linkage group (LG) 109 of the resistant parent CP95-1039 map, explaining 58% of the phenotypic variance with a logarithm of odds (LOD) of 19.3. The other two QTLs were located on LG 4 and LG 102 of the CP88-1762 map, explaining 12 and 8% of the phenotypic variance with a LOD of 5.4 and 3.4, respectively. Of the three QTLs, heterozygous genotypes of qORR109 and qORR102 positively contributed to orange rust resistance, whereas that of QTL qORR4 showed a negative contribution (**Table 3**). The three nearest markers, 2SNP350, 5SNPUN3354 and 3SNP3092, were 2 cM, 0.2 cM and 2.2 cM away from their corresponding QTL peak, qORR109, qORR4 and qORR102, respectively.

**Table 3:** Quantitative trait loci (QTL) associated with orange rust resistance of the F_1_ population derived from the cross CP95-1039 × CP88-1762.

Trait	QTL	LGs^a^	Position (cM)	LOD	Additive effect	PVE (%)^b^	Marker^c^	Distance (cM)^d^
Orange rust	qORR109	P1LG109	27.3	19.3	-2.07	58	2SNP350	2
Orange rust	qORR4	P2LG4	29.5	5.4	0.9	12	5SNPUN3354	0.2
Orange rust	qORR102	P2LG102	2.2	3.4	-0.67	8	3SNP3092	2.2

*^*a*^LG, Linkage group. ^*b*^PVE, Phenotypic variance explained. ^*c*^Nearest marker from the QTL peak. ^*d*^Distance of nearest marker from the respective QTL peak.*

To further study the genetic effect of the three QTLs, the phenotypic variation was dissected for each QTL and their combination (**Table 4**). Progeny containing the three susceptible QTL intervals based on the nearest marker alleles (Homozygote for QTL qORR109 and qORR102, and heterozygote for QTL qORR4) had a mean disease resistance score of 3.62, which served as the baseline for calculating orange rust disease reduction in the mapping population. With a single QTL, the disease severity can be reduced by 37.8 and 46.8% for QTLs qORR4 and qORR102, respectively. Since only one progeny existed in this category of single QTL qORR109 with heterozygote, the calculation of its contribution to rust resistance was ignored. With a combination of two QTLs, the disease severity could be reduced by 45.9, 34.3, and 19.6%, separately (**Table 4**). The combination of the three positive QTLs (Heterozygote for QTL qORR109 and qORR102, and homozygote for QTL qORR4) could reduce the disease severity by 56.4%.

**Table 4:** Phenotypic effects of quantitative trait loci (QTL) allele combinations on orange rust resistance in the F_1_ population from the cross CP95-1039 × CP88-1762.

qORR109,qORR4,qORR102^a^	Marker genotype^b^	No of progeny	Disease resistance (se)^c^	Reduction in disease severity (%)^d^
-,+,-	Ho.,He.,Ho.	12	3.62 ± 0.19	NA
+,+,-	He.,He.,Ho.	1	3.75	NA
-,-,-	Ho.,Ho.,Ho.	12	2.25 ± 0.29	37.8
-,+,+	Ho., He.,He.	29	2.91 ± 0.20	19.6
+,-,-	He.,Ho.,Ho.	49	1.96 ± 0.15	45.9
+,+,+	He.,He.,He.	12	2.38 ± 0.35	34.3
-,-,+	Ho.,Ho.,He.	19	1.93 ± 0.23	46.7
+,-,+	He.,Ho.,He.	18	1.58 ± 0.13	56.4

*^*a*^Symbols “+”and “-“represent presence and absence of corresponding QTL, respectively. ^*b*^Genotypes of the nearest marker for corresponding qORR109, qORR4, and qORR102. He., Heterozygous genotype. Ho., Homozygous genotype. ^*c*^Se, standard error. ^*d*^Reduction in disease severity compared with the mean severity of 12 progeny carrying three susceptible alleles.*

### Sugarcane SSRs and Putative *R* Genes

Out of 6,149 available SSR primers recruited from a literature search, a total of 1,095 sugarcane SSR primer pairs were uniquely aligned to the sorghum reference genome (Liu et al., unpublished data). Of all the mapped SSRs, 96 were found in the vicinity of three orange rust resistance QTL intervals and were close to the nearest markers of the QTLs according to the sorghum genome (Supplementary Table S2).

A custom-made sugarcane transcript database was formed including 700,194 sequences after removing redundant sequences. In total, 24,665 sugarcane transcripts were identified with orthologs in the sorghum genome. To identify sugarcane putative resistance genes, only the transcripts with orthologs in sorghum were utilized for subsequent analysis for a collinear comparison and application of the QTL intervals based on the sorghum genome. Out of the 24,665 sugarcane transcripts, 1,574 were characterized as putative *R* genes (Supplementary Table S3). Transcript sequences, corresponding sorghum orthologs, and reference *R* genes for sugarcane putative *R* genes were deposited in Supplementary Table S3.

### Developing Diagnostic Markers for MAS of Orange Rust Resistance in Sugarcane

Sugarcane SSRs in vicinity of the three orange rust resistance QTL intervals were used for diagnostic marker development. Of the 96 SSRs, only two showed robust polymorphisms between the two parental clones, CP95-1039 and CP88-1762. In addition, 61 gene-derived primer pairs crossing introns of 18 sugarcane putative *R* gene sequences were designed (Supplementary Table S2). Of the 61 pairs of *R* gene-derived primers, three showed polymorphisms between the two parental clones. The five polymorphic markers (two SSRs and three gene-derived) were first genotyped in the F_1_ mapping population. Interestingly, two gene-derived markers, G1 (*P* < 0.001) and M16 (*P* < 0.05), were significantly associated with orange rust resistance based on single marker analysis (**Table 5** and Supplementary Figure S2). The candidate diagnostic markers were further validated by genotyping 165 diverse sugarcane clones (Supplementary Table S1). The result further confirmed that G1 maker was associated with orange rust resistance (*P* < 0.05), while M16 was weakly associated. Based on the F_1_ mapping population, selection accuracy of G1 marker for orange rust resistance was 65.8% and the selection efficiency was 85.6%.

**Table 5:** Probability (*P*) value PCR-based markers on orange rust resistance.

	QTLs (% Exp.)	*P*-value for *t*-test	
		CP95-1039 × CP88-1762	Diversity CP clones
G1	qORR109 (58)	<0.001	0.044
M16	qORR4 (12)	0.011	0.092

## Discussion

Previously restricted to Asia, and the Pacific regions, sugarcane orange rust has also been detected in multiple countries in the western hemisphere (Comstock et al., 2008; Ovalle et al., 2008; Chavarria et al., 2009; Flores et al., 2009; Barbasso et al., 2010; Cadavid et al., 2012; Garcés et al., 2014). The outbreak of this disease could result in devastating economic loss, with an estimation of Aus$150–210 million in 2000 in Australia (Braithwaite et al., 2009). In Florida, orange rust has gained full attention due to its sudden emergence and negative impact on sugarcane production. In one crop season, economic losses associated with orange rust were estimated at $40 million (Dixon et al., 2010). It continues to be a serious disease for growers for several reasons. From a chemical perspective, optimal fungicide selection, and the rate, timing, and frequency of applications to prevent or control this newly emerged disease are unknown. Though several fungicides (e.g., strobilurin class fungicides and triazole class fungicides) have recently been registered and used to manage orange rust, more research is needed to fine-tune control recommendations that are economically reasonable (Rott et al., 2014). Biologically, orange rust pathogen releases a high density of spores into the air, and tolerates high temperatures, which allows the disease to develop throughout most of the sugarcane growing season and production areas, assuming infection conditions are suitable (Rott et al., 2014). Finally, the use of resistant host plants is currently limited because 77.5% of commercial cultivars grown in Florida and 41% of parental germplasm are susceptible to orange rust (Comstock et al., 2010). Unfortunately, it takes 12–14 years to release a new sugarcane cultivar with disease resistance, and even longer to propagate the cultivar for commercial production.

Compared to fungicide treatment, developing and using resistant cultivars is undoubtedly the thumbs-up approach for disease control because of a low input cost and healthy environment for sustainable sugarcane production. With reliable diagnostic markers developed, MAS would make this approach achievable for sugarcane breeding programs by quickly identifying resistant germplasm for crossing and selecting resistance progeny. In this study, we evaluated an F_1_ segregating population following a pseudo-testcross strategy for orange rust resistance QTL identification. Since the sugarcane is an outcrossing species and the parental clones for the F_1_ population were heterozygous, the F_1_ population was segregating genetically in the way similar as the backcross population of self-pollinating species if only the loci with homozygous genotype in one parent and heterozygous genotype in the other parent were considered. With multiple evaluation of the phenotype and high stringent genotypes of this segregating F_1_ population, we identified three QTLs controlling orange rust resistance for the first time, which can explain 58, 12, and 8% of the phenotypic variation, separately. Moreover, a PCR-based *R* gene-derived maker, G1 was developed, which showed significant association with orange rust resistance. The results of this research would be valuable for expediting release of resistant cultivars, thus contributing to the sustainable agriculture for orange rust disease control.

### QTL Analysis for Orange Rust Resistance

By performing QTL analysis using high density genetic maps and the mean scores of the eight rust resistance evaluations, for the first time we identified three QTLs controlling orange rust resistance. It’s noteworthy that GBS empowered QTL identification in sugarcane in this study. We used multiple SNP callers to maximize the detection of single dose markers, applied a robust filtering process on raw SNPs for each individual sample to improve SNP calling, and exploited a high LOD (≥10) in genetic map construction to avoid confusing linkage from different homoeologs (Yang et al., 2017b), which provide high density and reliable genetic maps for QTL analysis in this study. The three QTLs combined together could reduce 56.4% of disease severity compared with progeny containing the three susceptible QTL alleles (**Table 3**), indicating pyramiding these positive QTL alleles into sugarcane cultivars through MAS could effectively reduce the impact of the disease. So far, we have no evidence on whether these QTLs were linked to other disease resistance or agronomically important traits yet. The strategy for pyramiding multiple genes to improve disease resistance has been discussed in bean (Kelly et al., 1995), wheat (Liu et al., 2000), and rice (Singh et al., 2001; Jiang et al., 2004). Interestingly, two QTLs, qORR4 and qORR102, were identified from the same susceptible parental clone, CP88-1762, suggesting breeding materials that have robust agronomic traits but are susceptible to the disease should not directly be eliminated from sugarcane breeding programs as they may contain valuable minor resistance QTL alleles.

Although the molecular mechanisms of pathogen recognition and resistance to orange rust disease remain unclear, the resistance characterized in the current mapping population is less likely to be a single pathogen-race specific resistance since a mixture of orange rust spores collected from multiple highly susceptible cultivars from multiple fields were used to screen for resistance. Therefore, most likely a broad-based and durable resistance was identified within the segregating population. Although no genetic diversity has been reported in *P. kuehnii* so far, however, a change of orange rust pathogenicity has most likely been occurred in Florida. Earlier, major sugarcane cultivars CP88-1762 and CP89-2143 had been symptomless or resistant before 2010–2011, however, recently exhibited severe disease symptoms, suggesting a change in *P. kuehnii* pathogenicity (Comstock et al., 2010; Philippe Rott, Personal Communication). The resistance genes identified in the mapping population of this study may recognize multiple elicitors from orange rust pathogens, including pathogen-associated molecular patterns and/or weak effectors. Subsequent defense responses in sugarcane can be triggered to reduce the damage with contributing effects from each resistance gene. Since spores were artificially injected in the leaf, so lesions were also observed on leaves in the resistant type, but no sporulation was observed even after 4 weeks of infection (Supplementary Figure S1). The resistance in this mapping population may belong to a general non-specific resistance, in which sporulation of the fungus is inhibited to a low level to reduce the disease spread and severity (Kiraly et al., 2007). Therefore, this resistance should be fully exploited for breeding resistant sugarcane cultivars.

### Development of Diagnostic Markers for MAS of Orange Rust Resistance in Sugarcane

Release of orange rust resistant cultivars could be expedited by quickly identifying parental resistant germplasm and selecting resistant progeny through MAS using reliable molecular markers, especially PCR-based markers, which are easy to run for breeders without relying on fancy equipment and complicated procedure. In this study, we constructed a sugarcane putative *R* gene database (Supplementary Table S3) by bioinformatics analysis of several transcript databases. A total of 1,574 sugarcane putative *R* genes were annotated as NBS-LRR genes. Information on these putative *R* genes, with corresponding sorghum gene models, corresponding reference *R* genes, annotation, sugarcane transcript sequences (Supplementary Table S3) will be an important source to sugarcane research community for sugarcane disease resistance genes mining, genetic map construction, and PCR-based marker development.

In this study, we designed 61 *R* gene-derived primers according to the orange rust QTL intervals and the sugarcane putative *R* database. Out of the 61 primers, three (4.9%) showed polymorphisms with different fragment sizes between the two parental clones, indicating this strategy of crossing introns could functionally generate markers for subsequent analysis. Even more interestingly, after single marker analysis in the bi-parental mapping population and the diversity sugarcane clones, G1 marker was significantly linked to orange rust resistance, while M16 showed weak association (**Table 5**). We also screened 96 SSR markers; however, none of them was linked to orange rust resistance. Lack of linkage between the two polymorphic SSRs to orange rust resistance could be due to that the marker were far away from the QTL; or most likely that the marker linked with QTLs in a repulsion phase. The sugarcane genome is complex, with up to 12 sets of homo(eo)logs. A single locus may have multiple alleles and/or alleles with different dosages. Theoretically, only markers in coupling linkage with target QTLs contributing alleles can be used for MAS. Therefore, a successful rate of identifying PCR-based markers in the expected linkage is relatively low using this strategy.

Furthermore, we found that the target gene of G1 marker, *Sobic.002G166150*, is homologous to *RFO1*, a dominant *Arabidopsis* disease resistance gene, which encoded a wall-associated receptor-like kinase and conferred resistance to a broad spectrum of *Fusarium* races (Diener and Ausubel, 2005), further indicating that the resistance QTLs identified in this study carried durable instead of race-specific resistant genes. In this study, we used a mixture of orange rust spores collected from infected leaf of multiple sugarcane cultivars as inoculum (hard to culture this pathogen in medium). Thus the inoculum was most likely a mixture of multiple races, though the *Puccinia* race variation was not reported in North America yet. If the inoculum contained multiple races (we speculated that multiple races of *Puccinia* existed due to observation of the changing of host resistance over years; Comstock et al., 2010; Philippe Rott, Personal Communication), then resistance to the most popular orange rust pathogen races would be detected in our experiments. In another word, the QTLs we identified should harbor durable orange rust resistance gene(s) instead of a single race-specific resistance gene. The resistance provided by *RFO1* is quantitative and stronger resistance achieved if combined with other loci (additive effect). We also observed the same patterns in our results (**Table 4**). However, further experiments are needed to confirm whether sugarcane ortholog of *Sobic.002G166150* is the gene contributing to the orange rust resistance in sugarcane, and to elucidate the interactions among the three QTLs in sugarcane.

## Conclusion

QTL analysis revealed three QTLs controlling orange rust resistance, which can explain 58, 12, and 8% of the phenotypic variation, respectively. The three QTLs together can reduce 56.4% of disease severity compared with progeny containing the three susceptible QTL alleles. This is the first time that QTLs controlling orange rust resistance in sugarcane have been reported. To develop diagnostic markers for orange rust resistance, we identified 1,574 sugarcane putative *R* genes and aligned publicly available sugarcane SSRs to the sorghum genome. Out of 61 gene-derived markers and 96 SSRs screened, five were polymorphic with different fragment sizes between the two parental clones. The G1 marker was significantly linked to orange rust resistance based on single marker analysis. The putative QTLs and marker developed in this study can be utilized in sugarcane breeding programs to utilize MAS of orange rust resistance in sugarcane.

## Author Contributions

JW conceived and designed the experiments. XY, SS, MI, and SM performed the experiments. XY performed the data analysis. XY and JW drafted the manuscript. XY, JW, SS, SM, EH, MI, and JC revised the manuscript. All authors read and approved the final manuscript.

## Conflict of Interest Statement

The authors declare that the research was conducted in the absence of any commercial or financial relationships that could be construed as a potential conflict of interest.

## Footnotes

1 http://prgdb.org

2 http://biotools.umassmed.edu/bioapps/primer3_www.cgi
