# Supplementary material for: Identifying Quantitative Trait Loci (QTLs) and Developing Diagnostic Markers Linked to Orange Rust Resistance in Sugarcane (Saccharum spp.)
Source: Front Plant Sci. 2018 Mar 19;9:350. doi: 10.3389/fpls.2018.00350 (PMC5868124; doi:10.3389/fpls.2018.00350)
Supplement: Supplementary file 2 [file Image_1.PDF]

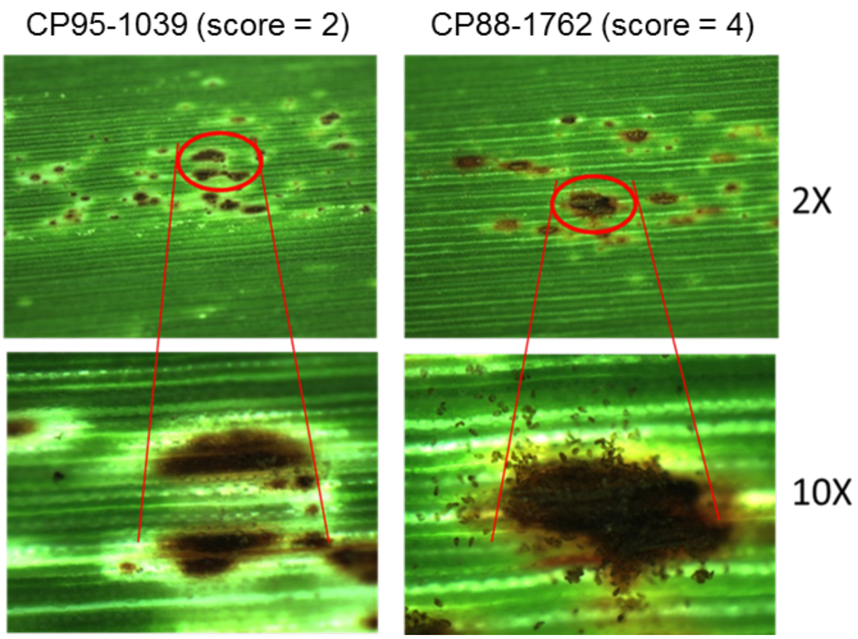

Figure S1 Symptoms of two parental lines after artificial inoculation under microscope.

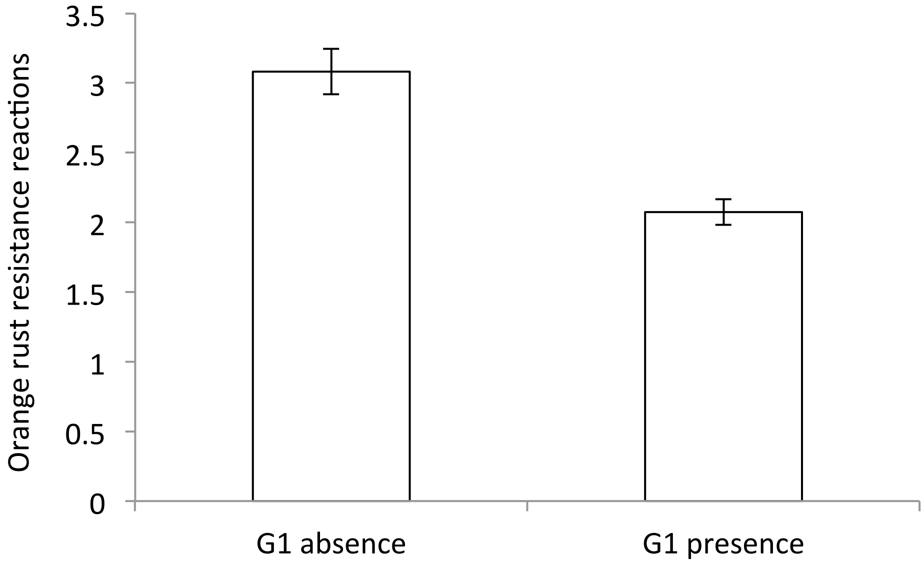

Figure S2 Orange rust resistance reactions evaluated for G1 marker in the F_1 mapping population.
